# Phenotypic heterogeneity in mycobacterial stringent response

**DOI:** 10.1186/1752-0509-5-18

**Published:** 2011-01-27

**Authors:** Sayantari Ghosh, Kamakshi Sureka, Bhaswar Ghosh, Indrani Bose, Joyoti Basu, Manikuntala Kundu

**Affiliations:** 1 Department of Physics, Bose Institute, Kolkata, India; 2 Department of Chemistry, Bose Institute, Kolkata, India; 3 Centre for Applied Mathematics and Computational Science, Saha Institute of Nuclear Physics, Kolkata, India

## Abstract

**Background:**

A common survival strategy of microorganisms subjected to stress involves the generation of phenotypic heterogeneity in the isogenic microbial population enabling a subset of the population to survive under stress. In a recent study, a mycobacterial population of *M*. *smegmatis *was shown to develop phenotypic heterogeneity under nutrient depletion. The observed heterogeneity is in the form of a bimodal distribution of the expression levels of the Green Fluorescent Protein (GFP) as reporter with the *gfp *fused to the promoter of the *rel *gene. The stringent response pathway is initiated in the subpopulation with high *rel *activity.

**Results:**

In the present study, we characterise quantitatively the single cell promoter activity of the three key genes, namely, *mprA*, *sigE *and *rel*, in the stringent response pathway with *gfp *as the reporter. The origin of bimodality in the GFP distribution lies in two stable expression states, i.e., bistability. We develop a theoretical model to study the dynamics of the stringent response pathway. The model incorporates a recently proposed mechanism of bistability based on positive feedback and cell growth retardation due to protein synthesis. Based on flow cytometry data, we establish that the distribution of GFP levels in the mycobacterial population at any point of time is a linear superposition of two invariant distributions, one Gaussian and the other lognormal, with only the coefficients in the linear combination depending on time. This allows us to use a binning algorithm and determine the time variation of the mean protein level, the fraction of cells in a subpopulation and also the coefficient of variation, a measure of gene expression noise.

**Conclusions:**

The results of the theoretical model along with a comprehensive analysis of the flow cytometry data provide definitive evidence for the coexistence of two subpopulations with overlapping protein distributions.

## Background

Microorganisms are subjected to a number of stresses during their lifetime. Examples of such stresses are: depletion of nutrients, environmental fluctuations, lack of oxygen, application of antibiotic drugs etc. Microorganisms take recourse to a number of strategies for survival under stress and adapting to changed circumstances [1-4]. A prominent feature of such strategies is the generation of phenotypic heterogeneity in an isogenic microbial population. The heterogeneity is advantageous as it gives rise to variant subpopulations which are better suited to persist under stress. Bistability refers to the appearance of two subpopulations with distinct phenotypic characteristics [5,6]. In one of the subpopulations, the expression of appropriate stress response genes is initiated resulting in adaptation. There are broadly two mechanisms for the generation of phenotypic heterogeneity [7,8]. In "responsive switching" cells switch phenotypes in response to perturbations associated with stress. In the case of "spontaneous stochastic switching", transitions occur randomly between the phenotypes even in the absence of stress. Responsive switching may also have a stochastic component as fluctuations in the level of a key regulatory molecule can activate the switch once a threshold level is crossed [1,3,5,6].

The pre-existing phenotypic heterogeneity, an example of the well-known "bet-hedging-strategy", keeps the population in readiness to deal with future calamities. Using a microfluidic device, Balaban et al. [9] have demonstrated the existence of two distinct subpopulations, normal and persister, in a growing colony of *E. coli *cells. The persister subpopulation constitutes a small fraction of the total cell population and is distinguished from the normal subpopulation by a reduced growth rate. Since killing by antibiotic drugs like ampicillin depends on the active growth of cell walls, the persister cells manage to survive when the total population is subjected to antibiotic treatment. The normal cells, with an enhanced growth rate are, however, unable to escape death. Once the antibiotic treatment is over, some surviving cells switch from the persister to the normal state so that normal population growth is resumed [9,10]. A simple theoretical model involving transitions between the normal and persister phenotypes explains the major experimental observations well [9,11]. In the case of environmental perturbations, Thattai and Oudenaarden [12] have shown through mathematical modeling that a dynamically heterogeneous bacterial population can under certain circumstances achieve a higher net growth rate conferring a fitness advantage than a homogeneous one. Mathematical modelling further shows that responsive switching is favoured over spontaneous switching in the case of rapid environmental fluctuations whereas the reverse is true when environmental perturbations are infrequent [7]. Another theoretical prediction that cells may tune the switching rates between phenotypes to the frequency of environmental changes has been verified in an experiment by Acar et al. [13] involving an engineered strain of S. cerevisiae which can switch randomly between two phenotypes. The major feature of all such studies is the coexistence of two distinct subpopulations in an isogenic population and their interconversions in the presence/absence of stress. Bistability, i.e., the partitioning of a cell population into two distinct subpopulations has been experimentally observed in a number of cases [1,3,5]. Some prominent examples include: lysis/lysogeny in bacteriophage λ [14], the activation of the lactose utilization pathway in *E. coli *[15] and the galactose utilization genetic circuit in S. cerevisiae [16], competence development in B. *subtilis *[6,17,18] and the stringent response in mycobacteria [19].

The mycobacterial pathogen *M*. *tuberculosis*, the causative agent of tuberculosis, has remarkable resilience against various physiological and environmental stresses including that induced by drugs [20-22]. On tubercular infection, granulomas form in the host tissues enclosing the infected cells. Mycobacteria encounter a changed physical environment in the confined space of granulomas with a paucity of life-sustaining constituents like nutrients, oxygen and iron [23,24]. The pathogens adapt to the stressed conditions and can survive over years in the so-called latent state. In vitro too, *M. tuberculosis *has been found to persist for years in the latent state characterised by the absence of active replication and metabolism [25]. Researchers have developed models simulating the possible environmental conditions in the granulomas. One such model is the adaptation to nutrient-depleted stationary phase [26]. The processes leading to the slowdown of replicative and metabolic activity constitute the stringent response. In mycobacteria, the expression of *rel *initiates the stringent response which leads to persistence. The importance of Rel arises from the fact that it synthesizes the stringent response regulator ppGpp (guanosine tetraphosphate) [27] and is essential for the long-term survival of *M. tuberculosis *under starvation [28] and for prolonged life of the bacilli in mice [29].

Key elements of the stringent response and the ability to survive over long periods of time under stress are shared between the mycobacterial species *M. tuberculosis *and *M. smegmatis *[30]. Recent experiments provide knowledge of the stress signaling pathway in mycobacteria linking polyphosphate (poly P), the two-component system MprAB, the alternate sigma factor SigE and Rel [31]. In an earlier study [19], we investigated the dynamics of *rel-gfp *expression (*gfp *fused with *rel *promoter) in *M. smegmatis *grown upto the stationary phase with nutrient depletion serving as the source of stress. In a flow cytometry experiment, we obtained evidence of a bimodal distribution in GFP levels and suggested that positive feedback in the stringent response pathway and gene expression noise are responsible for the creation of phenotypic heterogeneity in the mycobacterial population in terms of the expression of *rel-gfp*. Positive feedback gives rise to bistability [5,6], i.e., two stable expression states corresponding to low and high GFP levels. We further demonstrated hysteresis, a feature of bistability, in *rel-gfp *expression. The mathematical model developed by us to study the dynamics of the stringent response pathway predicted bistability in a narrow parameter regime which, however, lacks experimental support. In general, to obtain bistability a gene circuit must have positive feedback and cooperativity in the regulation of gene expression. Recently, Tan et al. [32] have proposed a new mechanism by which bistability arises from a noncooperative positive feedback circuit and circuit-induced growth retardation. The novel type of bistability was demonstrated in a synthetic gene circuit. The circuit, embedded in a host cell, consists of a single positive feedback loop in which the protein product X of a gene promotes its own synthesis in a noncooperative fashion. The protein decay rate has two components, the degradation rate and the dilution rate due to cell growth. In the circuit considered, production of X slows down cell growth so that at higher concentrations of X, the rate of dilution of X is reduced. This generates a second positive feedback loop since increased synthesis of X proteins results in faster accumulation of the proteins so that the protein concentration is higher. The combination of two positive feedback loops gives rise to bistability in the absence of cooperativity. A related study by Klumpp et al [33] has also suggested that cell growth inhibition by a protein results in positive feedback.

## Results

In this paper, we develop a theoretical model incorporating the effect of growth retardation due to protein synthesis [32,34]. We provide some preliminary experimental evidence in support of the possibility. In our earlier study [19], bimodality in the *rel-gfp *expression levels was observed. As a control, GFP expression driven by the constitutive *hsp60 *promoter was monitored as a function of time. A single bright population was observed at different times of growth (Figure S4 of [19]). The unimodal rather than bimodal distribution ruled out the possibility that clumping of mycobacterial cells and cell-to-cell variation of plasmid copy number were responsible for the observed bimodal fluorescence intensity distribution of *rel *promoter driven GFP expression. In the present study, we perform flow cytometry experiments to monitor *mprA-gfp *and *sigE-gfp *expression levels. The distribution of GFP levels in each case is found to be bimodal. We determine the probability distributions of the two subpopulations associated with low and high expression levels at different time points in the three cases of *mprA-gfp*, *sigE-gfp *and *rel-gfp *expression. In each case, the total distribution is a linear combination of two invariant distributions with the coefficients in the linear combination depending on time. The results of hysteresis experiments are also reported.

### Mathematical modeling of the stress response pathway

**Figure 1:**
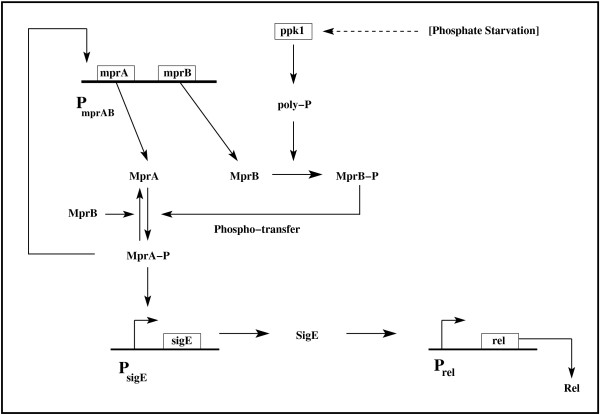
**Schematic diagram of the stringent response pathway in M. smegmatis activated under nutrient depletion**. MprB-P and MprA-P are the phosphorylated forms of MprB and MprA respectively. Poly P serves as the phosphate donor in the conversion of MprB to MprB-P.

Figure 1 shows a sketch of the important components of the stress response pathway in *M. smegmatis *subjected to nutrient depletion [19,31]. The operon *mprAB *consists of two genes *mprA *and *mprB *which encode the histidine kinase sensor MprB and its partner the cytoplasmic response regulator MprA respectively. The protein pair responds to environmental stimuli by initiating adaptive transcriptional programs. Polyphosphate kinase 1 (PPK1) catalyses the synthesis of polyphosphate (poly P) which is a linear polymer composed of several orthophosphate residues. Mycobacteria possibly encounter a phosphate-limited environment in macrophages. Sureka et al. [31] proposed that poly P could play a critical role under ATP depletion by providing phosphate for utilisation by MprAB. A recent experiment [34] on a population of *M. tuberculosis *has established that the MTB gene *ppk1 *is significantly upregulated due to phosphate starvation resulting in the synthesis of inorganic poliphosphate (poly P).

**Figure 2:**
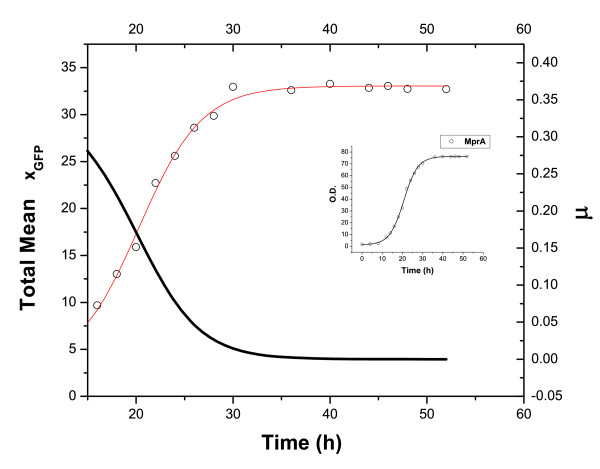
**Growth retardation due to protein synthesis**. (a) Mean amount of GFP fluorescence in the case of *mprA *promoter fused with *gfp *and (b) specific growth rate *μ *of mycobacterial population versus time in hours (h). The growth curve (optical density versus time) of the mycobacterial population is shown in the inset.

The two-component regulatory system SenX3-RegX3 is known to be activated on phosphate starvation in both *M. smegmatis *[35] and *M. tuberculosis *[34]. In the latter case, RegX3 has been shown to regulate the expression of *ppk1*, a feature expected to be shared by *M. smegmatis*. In both the mycobacterial populations, poly P regulates the stringent response via the *mprA-sigE-rel *pathway [31]. In our experiments, nutrient depletion possibly gives rise to phosphate starvation. On activation of the *mprAB *operon, MprB autophosphorylates itself with poly P serving as the phosphate donor [31,34]. The phosphorylated MprB-P phosphorylates MprA via phosphotransfer reactions. There is also evidence that MprB functions as a MprA-P (phosphorylated MprA) phosphatase. MprA-P binds the promoter of the *mprAB *operon to initiate transcription. A positive feedback loop is functional in the signaling network as the production of MprA brings about further MprA synthesis. The *mprAB *operon has a basal level of gene expression independent of the operation of the positive feedback loop. Once the *mprAB *operon is activated, MprA-P regulates the transcription of the alternate sigma factor gene *sigE*, which in turn controls the transcription of *rel*. We construct a mathematical model to study the dynamics of the above signaling pathway. The new feature included in the model takes into account the possibility that the production of stress-induced proteins like MprA and MprB slows down cell growth. This effectively generates a positive feedback loop as explained in Refs. [32,34]. Figure 2(a) shows the mean amount of GFP fluorescence in the total mycobacterial population as measured in a flow cytometry experiment (*mprA *promoter fused with *gfp*) versus time. Figure 2(a) shows the specific growth rate of the cell population versus time. The inset shows the experimental growth curve for the mycobacterial population. The growth was monitored by recording the absorbance values at 600 nm spectrophotometrically (see Methods). The specific growth rate at time *t *is given by $\frac{1}{N(t)}\frac{dN(t)}{dt}$ where *N*(*t*) is the number of mycobacterial cells at time *t*. Nutrient depletion limits growth and proliferation and culminates in the activation of stress response genes. It appears that in many cases rapid growth and stress response are mutually exclusive so that the production of a stress response protein gives rise to a slower growth rate [36]. The balance between the expressions of growth-related and stress-induced genes determines the cellular phenotype with respect to growth rate and stress response. Persister cells in both *E. coli *[9,10] and mycobacteria [21,22] have slow growth rates. In the case of *M. smegmatis*, we have already established that the slower growing persister subpopulation has a higher level of Rel, the initiator of stringent response, as compared to the normal subpopulation [19]. The new addition to our mathematical model [19] involves nonlinear protein decay rates arising from cell growth retardation due to protein synthesis. We briefly discuss the possible origins of the nonlinearity and its mathematical form [32,34]. The temporal rate of change of protein concentration is a balance between two terms: rate of synthesis and rate of decay. The decay rate constant (*γ_eff_*) has two components: the dilution rate due to cell growth (*μ*) and the natural decay rate constant (*γ*), i.e., *γ_eff _*= *μ *+ *γ *where *μ *is the specific growth rate. In many cases, the expression of a protein results in cell growth retardation [32,34]. The general form of the specific growth rate in such cases is given by

(1) $$\mu =\frac{\phi }{1+\theta x}$$

where *x *denotes the protein concentration and *ϕ*, *θ *are appropriate parameters. In Ref. [32], the expression for *μ *(Eq. (1)) is arrived at in the following manner. The Monod model [37] takes into account the effect of resource or nutrient limitation on the growth of bacterial cell population. The rate of change in the number of bacterial cells is

(2) $$\frac{dN}{dt}=\mu N$$

where the specific growth rate *μ *is given by

(3) $$\mu =\mu_{max}\frac{s}{k+s}$$

In (3), *s *is the nutrient concentration and *k *the half saturation constant for the specific nutrient. When *s *= *k*, the specific growth rate attains its half maximal value (*μ_max _*is the maximum value of specific growth rate). The metabolic burden of protein synthesis affecting the growth rate is modeled by reducing the nutrient amount *s *by *∈*, i.e.,

(4) $$\mu =\frac{\mu_{max}}{1+\frac{k}{s(1-\in )}}$$

The magnitude of is assumed to be small and proportional to the protein concentration *x*. Following the procedure outlined in the Supplementary Information of [32], namely, applying Taylor's expansion to (4) and putting *∈ *= λ*x *(λ is a constant), one obtains the expression in Eq. (1) with $\phi =\frac{\mu_{max}s}{s+k}$ and $\theta =\frac{k\lambda }{s+k}$.

**Figure 3:**
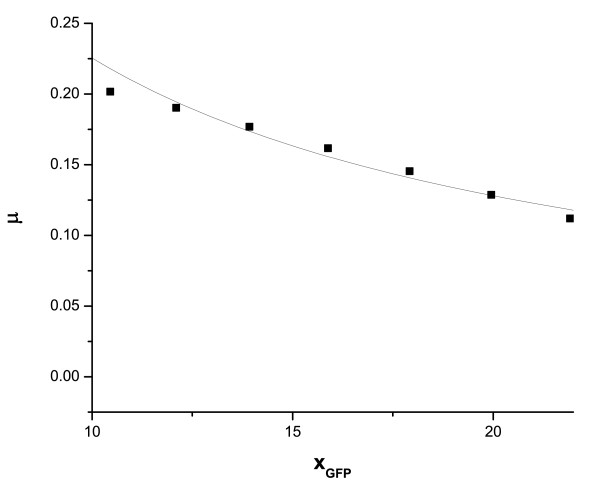
**Specific growth rate *μ *versus GFP fluorescence intensity *x_GFP _*fitted with an expression similar to that given in Eq. (1)**. The values of $\mu_{max}^{GFP}$ and *θ^GFP ^*are $\mu_{max}^{GFP}=0.94$ and *θ^GFP ^*= 0.317. The data points correspond to the growth period of 16-23 hours.

Thus, the decay rate of proteins has the form -*γ_eff _x *= -(*γ *+ *μ*)*x *where *μ *is given by Eq. (1). There are alternative explanations for the origin of the nonlinear decay term, e.g., the synthesis of a protein may retard cell growth if it is toxic to the cell [33]. In the case of mycobacteria, there is some experimental evidence of cell growth retardation brought about by protein synthesis. The response regulator MprA has an essential role in the stringent response pathway leading to persistence of mycobacteria under nutrient deprivation. Inactivation of the regulator in an *mprA *insertion mutant resulted in reduced persistence in a murine model but the growth of the mutant was proved to be significantly higher than that observed in the cases of the wild-type species [38,39]. Our experimental data (Figure 3) provide further support to the hypothesis that MprA synthesis leads to reduced specific growth rate. The data points represent GFP fluorescence intensity with *gfp *fused to the *mprA *promoter. The GFP acts as a reporter of the *mprA *promoter activity culminating in MprA (also MprB) synthesis. The data points shown in Figure 3 are those that correspond to the growth period of 16-23 hours in Figure 2.

The data points are fitted by an expression similar to that in Eq. (1) with $\mu_{max}^{GFP}=0.94$ and *θ^GFP ^*= 0.317. The differential equations describing the temporal rates of change of key protein concentrations in our model are described in the Additional File 1. Solving the equations, one finds the existence of bistability, i.e., two stable expression states in an extended parameter regime. Figures S1 A-C (Additional File 1) show the plots for bistability and hysteresis for the proteins MprA, SigE and Rel versus the autophosphorylation rate. In the deterministic scenario and in the bistable regime, all the cells in a population are in the same steady state if exposed to the same environment and with the same initial state. The experimentally observed heterogeneity in a genetically identical cell population is a consequence of stochastic gene expression. The biochemical events involved in gene expression are inherently probabilistic [40,41] in nature. The uncertainty introduces fluctuations (noise) around mean expression levels so that the single protein level of the deterministic case broadens into a distribution of levels. In the case of bistable gene expression, the distribution of protein levels in a population of cells is bimodal with two distinct peaks.

### Bimodal Expression of mprA, sigE and rel in M. smegmatis

**Figure 4:**
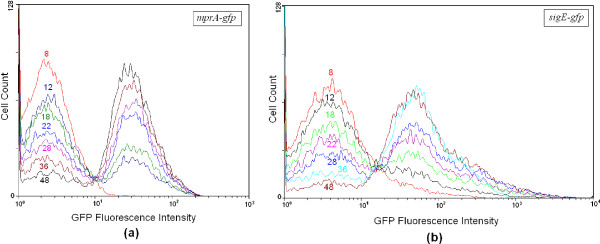
**Time course of (a) mprA-gfp and (b) sigE-gfp expression**. *M. smegmatis *harboring the appropriate promoter construct was grown for different periods of time (indicated in hours (h)) and the specific promoter-driven expression of GFP was monitored by flow cytometry. With time, there is a gradual transition from the L to the H subpopulation.

In the earlier study [19], we investigated the dynamics of *rel *transcription in individual cells of *M. smegmatis *grown in nutrient medium up to the stationary phase, with nutrient depletion serving as the source of stress. We employed flow cytometry to monitor the dynamics of green fluorescent protein (GFP) expression in *M. smegmatis *harboring the *rel *promoter fused to *gfp *as a function of time. The experimental signature of bistaility lies in the coexistence of two subpopulations. We now extend the study to investigate the dynamics of *mprA *and *sigE *transcription in individual *M. smegmatis *cells in separate flow cytometry experiments. Figure 4(a) and 4(b) show the time course of *mprA-*GFP and *sigE-*GFP expressions respectively as monitored by flow cytometry. In both the cases, the distribution of GFP-expressing cells is bimodal indicating the existence of two distinct subpopulations. In each case, the cells initially belong to the subpopulation with low GFP expression. The fraction of cells with high GFP expression increases as a function of time. The two subpopulations with low and high GFP expression are designated as L and H subpopulations respectively. In the stationary phase, the majority of the cells belong to the H subpopulation. The presence of two distinct subpopulations confirms the theoretical prediction of bistability.

We analysed the experimental data shown in Figure 4 and found that at any time point the distribution *P*(*x*, *t*) of GFP levels in a population of cells is a sum of two overlapping and time-independent distributions, one Gaussian (*P*_1_(*x*)) and the other lognormal (*P*_2_(*x*)), i.e.,

(5) $$P(x,t)=C_{1}(t)P_{1}(x)+C_{2}(t)P_{2}(x)$$

The coefficients *C_i_*'s (i = 1, 2) depend on time whereas *P*_1_(*x*) and *P*_2_(*x*) are time-independent. The general forms of *P*_1_(*x*) and *P*_2_(*x*) are,

(6) $$P_{1}(x)=\frac{exp\left(-{\left(\frac{x-x_{01}}{w_{01}}\right)}^{2}\right)}{w_{01}\sqrt{\frac{\pi }{2}}}$$

(7) $$P_{2}(x)=\frac{exp\left(-\frac{1}{2}{\left(\frac{lnx-x_{02}}{w_{02}}\right)}^{2}\right)}{xw_{02}\sqrt{2\pi }}$$

**Figure 5:**
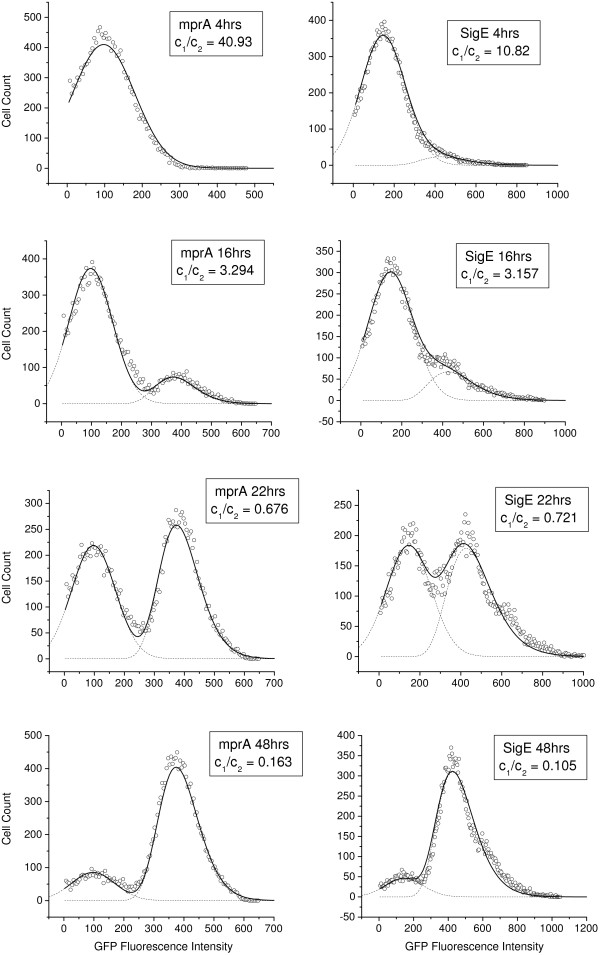
**Fitting of data with two distributions**. Experimental data for cell count versus GFP fluorescence intensity at selected time points when *gfp *is fused with *mprA *and *sigE *promoters respectively. The solid curve represents *P *(*x*, *t*) in equation (5) and the dotted curves are the individual terms on the r.h.s.

Figures S2(a) and (b) in Additional File 1 illustrate the typical forms of the Gaussian and lognormal distributions. The Gaussian distribution has a symmetric form whereas the lognormal distribution is asymmetric and long-tailed. Figure 5 shows the experimental data for cell count versus GFP fluorescence intensity at selected time points in the cases when *gfp *is fused with *mprA *and *sigE *promoters in separate experiments. The dotted curves represent the individual terms in the r.h.s. of Eq. (5) and the solid curve denotes the linear combination *P*(*x*, *t*). The different parameters of *P*_1_(*x*) and *P*_2_(*x*) have the values *x*_01 _= 97.3366 (145.86181); *w*_01 _= 103.0731 (154.67381); *x*_02 _= 5.95526 (6.1171); *w*_02 _= 0.17618 (0.2509) when *gfp *is fused with *mprA *(*sigE*). The ratio of the coefficients, *C*_1_(*t*)/*C*_2_(*t*), has the value listed by the side of each figure. Figure S3 displays a similar analysis of the experimental data when *gfp *is fused to the *rel *promoter.

In the earlier study [19], the total cell population was divided into L and H subpopulations depending on whether the measured GFP fluorescence intensity was less or greater than a threshold intensity. In the present study, we have obtained approximate analytic expressions for the distributions of GFP fluorescence intensity in the L and H subpopulations. The two distributions, Gaussian and lognormal, have overlaps in a range of fluorescence intensity values (Figure 5 and Figure S3 in Additional File 1). We next used the binning algorithm developed by Chang et al. [42] to partition the cells of the total population into two overlapping distributions, one Gaussian (Eq. (6)) and the other lognormal (Eq. (7)). At time t, let N(t) be the total number of cells. For each cell, the data *x_j _*for the fluorescence intensity is used to calculate the ratios,

(8) $$g_{1}(x_{j})=\frac{P_{1}(x_{j})}{P_{1}(x_{j})+P_{2}(x_{j})},\;\;\;g_{2}(x_{j})=\frac{P_{2}(x_{j})}{P_{1}(x_{j})+P_{2}(x_{j})}$$

where *P*_1_(*x*) and *P*_2_(*x*) are the distributions in Eqs. (6) and (7). A random number r is generated and the cell *j *is assigned to the L subpopulation if 0 ≤ *r *<*g*_1_(*x_j_*), the cell belongs to the H subpopulation otherwise. Once the total population is partitioned into the L and H subpopulations, one can calculate the following quantities:

(9) $$\begin{array}{l} \omega_{i}(t)=\frac{N_{i}(t)}{N(t)}\;\;\;\;(i=1,2)\\ \mu_{i}(t)=\sum x_{ji}(t)/N_{i}(t)\\ \sigma_{i}^{2}(t)=\sum {\left(x_{ji}(t)\;-\;\mu_{i}(t)\right)}^{2}/(N_{i}(t)-1) \end{array}$$

**Figure 6:**
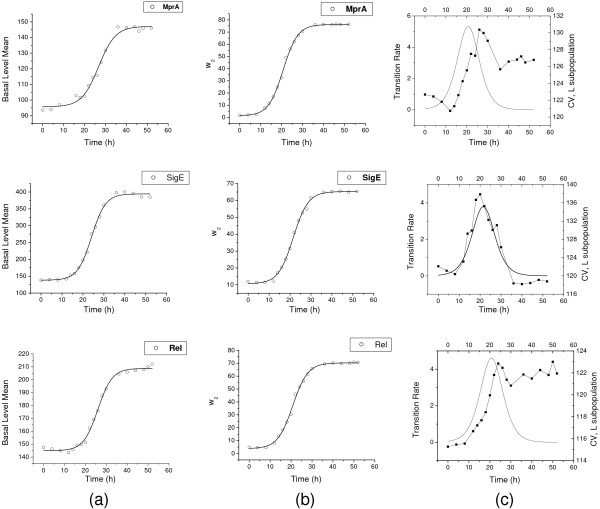
**Analysis of the time course of gfp expression**. (a) Mean protein level in L subpopulation (basal level) versus time in hours in the three cases of *gfp *fused with *mprA*, *sigE *and *rel *promoters respectively. (b) Fraction of cells *ω*_2_(t) in the H subpopulation versus time in hours in the three cases. (c) Transition rate from the L to the H subpopulation and the CV (experimental data shown) of the protein levels in the L subpopulation versus time in hours in the three cases. The experimental data are analyzed using the binning algorithm to obtain the plots (a), (b) and (c).

The indices *i *= 1, 2 correspond to the L and H subpopulations, *ω_i_*(*t*) is the fraction of cells in the *i*th subpopulation at time t, *μ_i_*(*t*) is the mean fluorescence intensity for the *i*th subpopulation and $\sigma_{i}^{2}(t)$ the associated variance. Figure 6 shows the results of the data analysis. Figure 6(a) shows the plots of mean GFP fluorescence level for the L subpopulation (basal level) versus time in the three cases of *gfp *fused with the promoters of *mprA*, *sigE *and *rel *respectively. Figure 6(b) displays the data for the fractions of cells, ω_2_(*t*), versus time in the three cases and Figure 6(c) shows the transition rate versus time along with the coefficients of variation CV (CV = standard deviation/mean) of the protein levels in the L subpopulation versus time.

Figure S4 (Additional File 1) shows the plots of mean GFP fluorescence level for the total population versus time in the three cases of *gfp *fused with the promoters of *mprA*, *sigE *and *rel *respectively. As in the case of the basal level versus time data (Figure 6(a)), the plots are sigmoidal in nature. We solved the differential equations of the theoretical model described in Additional File 1 and obtained the concentrations of MprA, MprB, SigE, MprA-P, MprB-P and GFP versus time. Some of these plots are shown in Figure S5 (Additional File 1) and reproduce the sigmoidal nature of the experimental plots. We note that the sigmoidal nature of the curves is obtained only when the non-linear nature of the degradation rate is taken into account.

As we have already discussed, the distribution of GFP levels in the mycobacterial cell population is a linear combination of two invariant distributions, one Gaussian and the other lognormal, with only the coefficients in the linear combination dependent on time. Friedman et al. [43] have developed an analytical framework of stochastic gene expression and shown that the steady state distribution of protein levels is given by the gamma distribution. The theory was later extended to include the cases of transcriptional autoregulation as well as noise propagation in a simple genetic network. While experimental support for gamma distribution has been obtained earlier [44], a recent exhaustive study [45] of the *E. coli *proteome and transcriptome with single-molecule sensitivity in single cells has established that the distributions of almost all the protein levels out of the 1018 proteins investigated, are well fitted by the gamma distribution in the steady state. The gamma distribution was found to give a better fit than the lognormal distribution for proteins with low expression levels and almost similar fits for proteins with high expression levels. We analysed our GFP expression data to compare the fits using lognormal and gamma distributions. For all the three sets of data (*gfp *fused with the promoters of *mprA*, *sigE *and *rel*), the lognormal and gamma distribution give similar fits at the different time points. Figure S6 (Additional File 1) shows a comparison of the fits for the case of *gfp*-*mprA*. The lognormal appears to give a somewhat better fit than the gamma distribution, specially at the tail ends.

### Hysteresis in gfp expression

**Figure 7:**
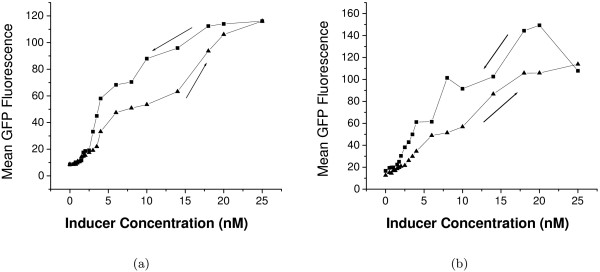
**Hysteresis in gfp expression**. The gene *gfp *is fused with (a) *mprA *and (b) *sigE *promoter. Filled triangles and squares represent the experimental data of mean GFP fluorescence with increasing and decreasing concentrations of tetracycline inducer respectively.

**Figure 8:**
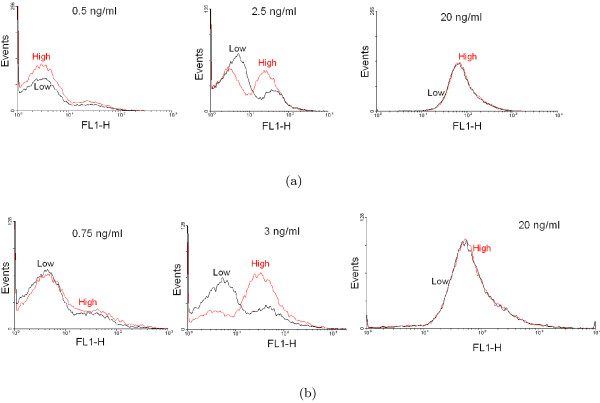
**Hysteresis via GFP distributions**. The distributions in the stationary phase with two different histories (see text) when *gfp *is fused with (a) *mprA *and (b) *sigE *promoter. The specific inducer concentrations are mentioned with each plot.

Some bistable systems exhibit hysteresis, i.e., the response of the system is history-dependent. In the earlier study, experimental evidence of hysteresis was obtained with *gfp *fused to the promoter of *rel*. The experimental procedure followed for the observation of hysteresis is as follows. In PPK-KO, the *ppk1 *knockout mutant, the *ppk1 *gene was introduced under the control of the *tet *promoter. We grew PPK-KO carrying the tetracycline-inducible *ppk1 *and *rel-gfp *plasmid in medium with increasing concentration of tetracycline (inducer). For each inducer concentration, the distribution of cells expressing *gfp *was analysed by flow cytometry in the stationary phase (steady state) and the mean GFP level was measured. A similar set of experiments was carried out for decreasing concentrations of tetracycline. In the present study, hysteresis experiments in the manner described above were carried out in the two cases of *gfp *fused to *mprA *and *sigE *promoters respectively. Figure 7 shows the hysteresis data (mean GFP fluorescence versus inducer concentration) in the two cases for increasing (branch going up) and decreasing (branch going down) inducer concentrations. The existence of two distinct branches is a confirmation of hysteresis in agreement with theoretical predictions (Figures S1 A-C). Figure 8 shows the GFP distributions in the stationary phase for two sets of experiments with different histories, one in which the inducer concentration is increased from low to a specific value (indicated as "Low" in black) and the other in which the same inducer concentration is reached by decreasing the inducer concentration from a high value (indicated as "High" in red). The distributions show that two regions of monostability are separated by a region of bistability. In the cases of monostability, the distributions with different histories more or less coincide. In the region of bistability, the distributions are distinct indicating a persistent memory of initial conditions.

## Discussion

The development of persistence in microbial populations subjected to stress has been investigated extensively in microorganisms like *E. coli *and mycobacteria [9,10,21,22,28,29]. In an earlier study [19], we demonstrated the roles of positive feedback and gene expression noise in generating phenotypic heterogeneity in a population of *M. smegmatis *subjected to nutrient depletion. The heterogeneity was in terms of two distinct subpopulations designated as L and H subpopulations. The subpopulations corresponded to persister and non-persister cell populations with the stringent response being initiated in the former. In the present study, we have undertaken a comprehensive single cell analysis of the expression activity of the three key molecular players in the stringent response pathway, namely, MprA, SigE and Rel. This has been done by fusing *gfp *to the respective genes in separate experiments and monitoring the GFP levels in a population of cells via flow cytometry. The distribution has been found to be bimodal in each case.

In our earlier study [19], with only the positive autoregulation of the *mprAB *operon taken into account, bistability was obtained in a parameter regime with restricted experimental relevance. The inclusion of the effective positive feedback loop due to growth retardation by protein synthesis gives rise to a considerably more extended region of bistability in parameter space. The persister cells with high stringent response regulator levels are known to have slow growth rates [21,22,28,29]. This is consistent with the view that stress response diverts resources from growth to stress-related functions resulting in the slow growth of stress-resistant cells [36]. Figure 2 and 3 provide experimental evidence that the mean intensity of GFP fluorescence monitoring *mprA-gfp *expression increases with time while the specific growth rate *μ *of the *M*. *smegmatis *population decreases in the same time interval. The reciprocal relationship between the two quantities is represented by an expression similar to that in Eq. (1). Since our knowledge of the detailed genetic circuitry involved in the stringent response is limited, we have not attempted to develop a model to explain the origin of cell growth retardation due to protein synthesis. Further experiments (e.g., sorting of the mycobacterial cell population into two subpopulations) are needed to provide conclusive evidence that increased protein synthesis retards cell growth. The stringent response pathway involving MprA and MprB is initiated when the mycobacterial population is subjected to stresses like nutrient depletion. There is now experimental evidence of complex transcriptional, translational, and posttranslational regulation of SigE in mycobacteria [46-49]. A double positive feedback loop arises due to the activation of transcription initiation of *sigE *by MprA-P and the activation of the transcription of the *mprAB *operon by the SigE-RNAP complex. Posttranslational regulation of SigE is mediated by RseA, an anti-sigma factor.

Barik et al. [49] have identified a novel positive feedback involving SigE and RseA which becomes functional under surface stress. More experiments need to be carried out to obtain insight on the intricate control mechanisms at work when mycobacteria are subjected to stresses like nutrient deprivation. This will lead to a better understanding of the major contributory factors towards the generation of phenotypic heterogeneity in mycobacterial populations subjected to stress.

## Conclusions

In the present study, we have characterised quantatively the single cell promoter activity of three key genes in the stringent response pathway of the mycobacterial population *M. smegmatis*. Under nutrient depletion, a "responsive switching" occurs from the L to the H subpopulation with low and high expression levels respectively. A comprehensive analysis of the flow cytometry data demonstrates the coexistence of two subpopulations with overlapping protein distributions. We have further established that the GFP distribution at any time point is a linear superposition of a Gaussian and a lognormal distribution. The coefficients in the linear combination depend on time whereas the component distributions are time-invariant. The Gaussian and lognormal distributions describe the distribution of protein levels in the L and H subpopulations respectively. The two distributions overlap in a range of GFP fluorescence intensity values. We also find that the experimental data for the H subpopulation can be fitted very well by the gamma distribution though the lognormal distribution gives a slightly better fit. In the case of skewed positive data sets, the two distributions are often interchangeable [50]. An analytical framework similar to that in Ref. [43] is, however, yet to be developed for the mycobacterial stringent response pathway studied in the paper. The major components in the pathway are the two-component system *mprAB *and multiple positive feedback loops. The two-component system is known to promote robust input-output relations [51] and persistence of gene expression states [52] which may partly explain the good fitting of the experimental data by well-known distributions. Further quantitative measurements combined with appropriate stochastic modeling are needed to characterise the experimentally observed subpopulations more uniquely. We used the binning algorithm developed in [42] to partition the experimental cell population into the L and H subpopulations. This enabled us to compute quantities like the mean protein level in the L subpopulation, the fraction of cells in the H subpopulation and the CV of GFP levels in the L subpopulation as a function of time. The picture that emerges from the analysis of experimental data is that of bistability, i.e., the coexistence of two distinct subpopulations and stochastic transitions between the subpopulations resulting in the time evolution of the fraction of cells in the H subpopulation. As pointed out in the earlier study [19], the rate of transition to the H subpopulation and the CV of the L subpopulation levels attain their maximum values around the same time point (Figure 5(c)) indicating the role of gene expression noise in bringing about the transition from the L to the H subpopulation. We have not attempted to develop theoretical models describing the time evolution of the relative weights, *ω_i_*'s (*i *= 1, 2), of the two subpopulations (Eq. (9)). A simple model of two interacting and evolving subpopulations with linear first order kinetics [12], cannot explain the sigmoidal nature of the time evolution. A model with nonlinear growth kinetics has been proposed in [42] but lacking definitive knowledge on the origin of nonlinearity in the growth of mycobacterial subpopulations we defer the task of model building to a future publication.

## Methods

### Strains

*M. smegmatis *mc^2^155 was grown routinely in Middle Brook (MB) 7H9 broth (BD Biosciences) medium supplemented with 2% glucose and 0.05% Tween 80.

### Construction of plasmids for fluorescence measurements

The *mprAB *promoter was amplified from the genomic DNA of *M. smegmatis *using the sense and antisense primers, 5'-AA**GGTACC**GCGCAACACCACAAAAAGCG-3' and 5'-TA**GGATCC**AGTTTTGACTCACTATCTGAG-3' respectively and cloned into the promoter-less replicative *gfp *vector pFPV27 between the KpnI and BamHI sites (in bold). The *sigE *and *rel *promoters fused to *gfp *have been described earlier [19,31]. The resulting plasmids were electroporated into *M. smegmatis *mc^2^155 for further study. For the study of hysteresis, expression of *ppk1 *under a tetracycline-inducible promoter in an *M. smegmatis *strain inactivated in the *ppk1 *gene (PPK-KO), has been described earlier [19].

### FACS analysis

*M. smegmatis *cells expressing different promoters fused to GFP were grown in medium supplemented with kanamycin (25 *μg*/*ml*) and analysed at different points of time on a FACS Caliber (BD Biosciences) flow cytometer as described earlier [19]. Briefly, cells were washed, resuspended in PBS and fluorescence intensity of 20,000 events was measured. The data was analyzed using Cell Quest Pro (BD Biosciences) and WINMIDI software. The flow cytometry data is represented in histogram plots where the x-axis is a measure of fluorescence intensity and the y-axis represents the number of events.

### Measurement of growth rate

*M. smegmatis *expressing promoter-*gfp *fusion constructs were grown in Middle Brook (MB) 7H9 broth supplemented with glucose and Tween 80, and kanamycin (25 *μg*/*ml*). Growth at different time points was measured by recording absorbance values at 600 nm (a value of 1 OD_600 _is equal to 10^8 ^cells or 200 *μg *dry weight of cells). A growth curve was generated by plotting absorbance values against time (inset of Figure 2). The specific growth rate *μ *(Eq. (2)) at different time points is determined by taking derivatives of the growth curve at the different time points (Figure 2).

## Authors' contributions

IB, JB and MK conceptualised, supervised and coordinated the study. KS, JB and MK carried out the experiments. SG, BG and IB developed the theoretical model, performed the data analysis and interpreted the data. IB drafted the manuscript. All authors read and approved of the final version.

## Supplementary Material

Additional file 1 **Supplementary Information**. Contains description of mathematical model including Figures S1-S6. Click here for file
