# Supplementary material for: Phenotypic heterogeneity in mycobacterial stringent response
Source: BMC Syst Biol. 2011 Jan 27;5:18. doi: 10.1186/1752-0509-5-18 (PMC3045321; doi:10.1186/1752-0509-5-18)
Supplement: Additional file 1 — Supplementary Information. Contains description of mathematical model including Figures S1-S6. [file 1752-0509-5-18-S1.PDF]

Supplementary Information

Phenotypic Heterogeneity in Mycobacterial Stringent Response

Sayantari Ghosh[1], Kamakshi Sureka[2], Bhaswar Ghosh[3], Indrani Bose[1*],
Joyoti Basu[2], Manikuntala Kundu[2]

1 Department of Physics, Bose Institute, Kolkata, India

2 Department of Chemistry, Bose Institute, Kolkata, India

3 Centre for Applied Mathematics and Computational Science , Saha Institute of Nuclear Physics, Kolkata, India

Contents

I **Description of mathematical model**

II **Figures S1-S6**

Mathematical model

The reaction scheme describing the processes shown in Figure 1 is given by

$$B_2 + B_2 \underset{k_2}{\overset{k_1}{\rightleftharpoons}} B_2 - B_2 \xrightarrow{k_3} B_1 + B_1 \tag{1}$$

$$A_2 + B_1 \underset{k_5}{\overset{k_4}{\rightleftharpoons}} A_2 - B_1 \xrightarrow{k_6} A_1 + B_2 \tag{2}$$

$$A_1 + B_2 \underset{k_8}{\overset{k_7}{\rightleftharpoons}} A_1 - B_2 \xrightarrow{k_9} A_2 + B_2 \tag{3}$$

$$G_{AB} + A_1 \underset{k_d}{\overset{k_a}{\rightleftharpoons}} G^*_{AB} \xrightarrow{\beta} A_2 + B_2 \tag{4}$$

$$G_{AB} + A_1 \xrightarrow{k_s} A_2 + B_2 \tag{5}$$

In the equations, $A_1(A_2)$ represents the phosphorylated (unphosphorylated) form of MprA and $B_1(B_2)$ denotes the phosphorylated (unphosphorylated) form of MprB. The inactive and active states of the *mprAB* operon are represented by G_{AB} and ${G_{AB}}^*$ respectively. In the inactive state, MprA and MprB proteins are synthesized at a basal rate k_s and in the active state protein production occurs at an enhanced rate β. Eq. (1) describes the autophosphorylation reaction of MprB with the poly P chain serving as a source of phosphate groups. Eq. (2) describes the transfer of the phosphate group from the phosphorylated MprB to MprA. Eq. (3) corresponds to dephosphorylation of phosphorylated MprA by unphosphorylated MprB which thus acts as a phosphatase (in the earlier study [1], phosphorylated MprB was assumed to act as a phosphatase which is not consistent with experimental evidence). Eqs. (4) and (5) describe activation of the *mprAB* operon by phosphorylated MprA and basal expression of the operon respectively. Refs. [2-4] provide experimental justification for the reaction scheme shown in Eqs. (1)-(5). Using standard mass action kinetics, we write down the rate equations for the concentration of each of the key molecular species participating in the biochemical events. The equations are:

$$\frac{d[A_1]}{dt} = k_6[A_2 - B_1] - k_7[A_1][B_2] + k_8[A_1 - B_2] - \gamma[A_1] - \frac{\phi[A_1]}{1 + \theta_1[A_1]} \tag{6}$$

$$\frac{d[A_2]}{dt} = k_s + \beta\frac{[A_1]/k}{1 + [A_1]/k} + k_9[A_1 - B_2] - k_4[A_2][B_1] + k_5[A_2 - B_1] - \gamma_1[A_2] - \frac{\phi[A_2]}{1 + \theta_2[A_2]} \tag{7}$$

$$\frac{d[B_1]}{dt} = k_3[B_2 - B_2] - k_4[A_2][B_1] + k_5[A_2 - B_1] - \gamma[B_1] - \frac{\phi[B_1]}{1 + \theta_1[B_1]} \tag{8}$$

$$\frac{d[B_2]}{dt} = k_s + \beta\frac{[A_1]/k}{1 + [A_1]/k} + k_2[B_2 - B_2] - k_1[B_2]^2 + k_6[A_2 - B_1] - k_7[A_1][B_2] - \gamma[B_2] - \frac{\phi[B_2]}{1 + \theta_2[B_2]} \tag{9}$$

$$\frac{d[B_2 - B_2]}{dt} = -k_2[B_2 - B_2] + k_1[B_2]^2 - k_3[B_2 - B_2] + (k_8 + k_9)[A_1 - B_2] \tag{10}$$

$$\frac{d[A_2 - B_1]}{dt} = k_4[A_2][B_1] - k_5[A_2 - B_1] - k_6[A_2 - B_1] \tag{11}$$

$$\frac{d[A_1-B_2]}{dt} = k_7[A_1][B_2]-(k_8+k_9)[A_1-B_2] \tag{12}$$

$$\frac{d[SigE]}{dt} = s_1+\beta_1\frac{[A_1]/k'}{1+[A_1]/k'}-\delta_1[SigE] \tag{13}$$

$$\frac{d[GFP]}{dt} = s_2+\beta_2\frac{[SigE]/k''}{1+[SigE]/k''}-\delta_2[GFP] \tag{14}$$

Eq. (13) represents SigE synthesis due to transcriptional activation of the *sigE* gene by phosphorylated MprA-P. Eq. (14) describes GFP production due to the activation of the *rel* promoter by SigE. The rate constants γ, δ_1 and δ_2 are the degradation rate constants. The last terms in Eqs. (6)-(9) represent the nonlinear decay rates the genesis of which is explained in the main text (see Eq. 4) [5]. Eqs. (6)-(14) correspond to the case where *gfp* is fused to the *rel* promoter. In the other cases when *gfp* is fused to the *mprA* or *sigE* promoter, appropriate modifications in the set of equations are required.

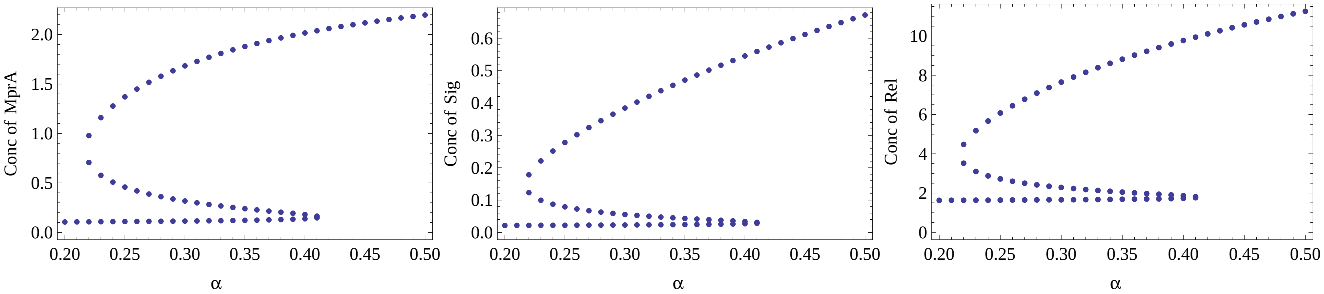

Figure S1: Bistability and hysteresis in the deterministic model. Steady state concentrations of MprA, SigE and Rel versus the parameter α (Eq. (21)). With [c] as the unit of concentration, say, nanomolar (nm) the units of x and y axis variables are $[c]^{-1}[t]^{-1}$ and $[c]$ respectively.

The steady state solution of Eqs. (6)-(14) is obtained by setting all the rates of change to be zero. In the case of bistability, there are three steady state solutions, two stable and one unstable [6,7,8]. In the steady state, one has to solve the following set of coupled nonlinear algebraic equations:

$$\alpha_1[A_2][B_1]-\alpha_2[A_1][B_2]-\gamma[A_1]-\frac{\phi[A_1]}{1+\theta_1[A_1]}=0 \tag{15}$$

$$k_s+\beta\frac{[A_1]/k}{1+[A_1]/k}-\alpha_1[A_2][B_1]+\alpha_2[A_1][B_2]-\gamma_1[A_2]-\frac{\phi[A_2]}{1+\theta_2[A_2]}=0 \tag{16}$$

$$\alpha[B_2]^2-\alpha_1[A_2][B_1]-\gamma[B_1]-\frac{\phi[B_1]}{1+\theta_1[B_1]}=0 \tag{17}$$

$$k_s+\beta\frac{[A_1]/k}{1+[A_1]/k}-\alpha[B_2]^2+\alpha_1[A_2][B_1]-\gamma[B_2]-\frac{\phi[B_2]}{1+\theta_2[B_2]}=0 \tag{18}$$

$$s_1+\beta_1\frac{[A_1]/k'}{1+[A_1]/k'}-\delta_1[SigE]=0 \tag{19}$$

$$s_2+\beta_2\frac{[SigE]/k''}{1+[SigE]/k''}-\delta_2[GFP]=0 \tag{20}$$

where,

$$\alpha=\frac{k_1k_3}{k_2+k_3},\ \alpha_1=\frac{k_4k_6}{k_5+k_6},\ \alpha_2=\frac{k_7k_9}{k_8+k_9},\ k=\frac{k_d}{k_a} \tag{21}$$

The solutions of Eqs. (15)-(20) are obtained with the help of Mathematica. Figures S1 A-C show the steady state solutions generated by varying the parameter α (associated with the autophosphorylation of MprB). The parameters have values: $\alpha_1 = 2.4, \alpha_2 = 2.8, \gamma = 0.1, k_s = 0.14, \beta = 4, k = 1, \gamma_1 = 1, \phi = 0.5, \theta_1 = 1, \theta_2 = 10, s_1 = 0.02, \beta_1 = 4, k' = 10, \delta_1 = 1,\ s_2 = 0.12, \beta_2 = 4, k'' = 2, \delta_2 = 0.1$ in appropriate units.

In each of the Figures S1 A-C, the solid and dotted branches represent stable and unstable steady states respectively. Bistability is obtained over a wide range of parameter values due to the inclusion of the non-linear decay terms in Eqs. (6)-(9). In the hysteresis experiments, the inducer tetracycline was used to control the level of PPK1 and therefore the synthesis of the poly P chain. Since the latter acts as the source of phosphate groups for the autophosphorylation of MprB (Eq. (1)), the rate constant k_1, in Eq. (1) is effectively proportional to the inducer (or the PPK1) concentration. Since the parameter α (Eq. (21)) includes the rate constant k_1, a varying inducer concentration is equivalent to varying the parameter α. There is some experimental evidence that MprA-P regulates the expression of the *mprAB* operon in the form of dimers [9]. Inclusion of this feature in our model makes the bistable behaviour more prominent.

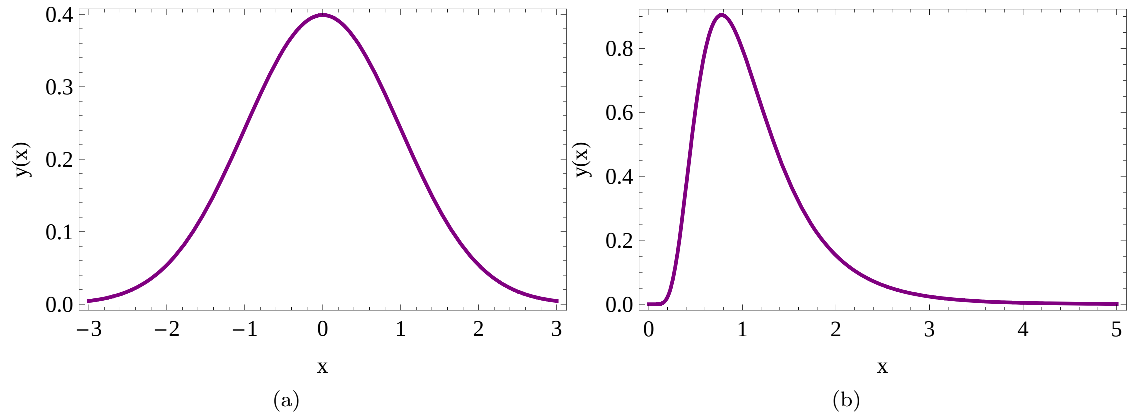

Figure S2: (a) Gaussian and (b) lognormal distributions which describe the distribution of GFP levels in the L and H subpopulations respectively.

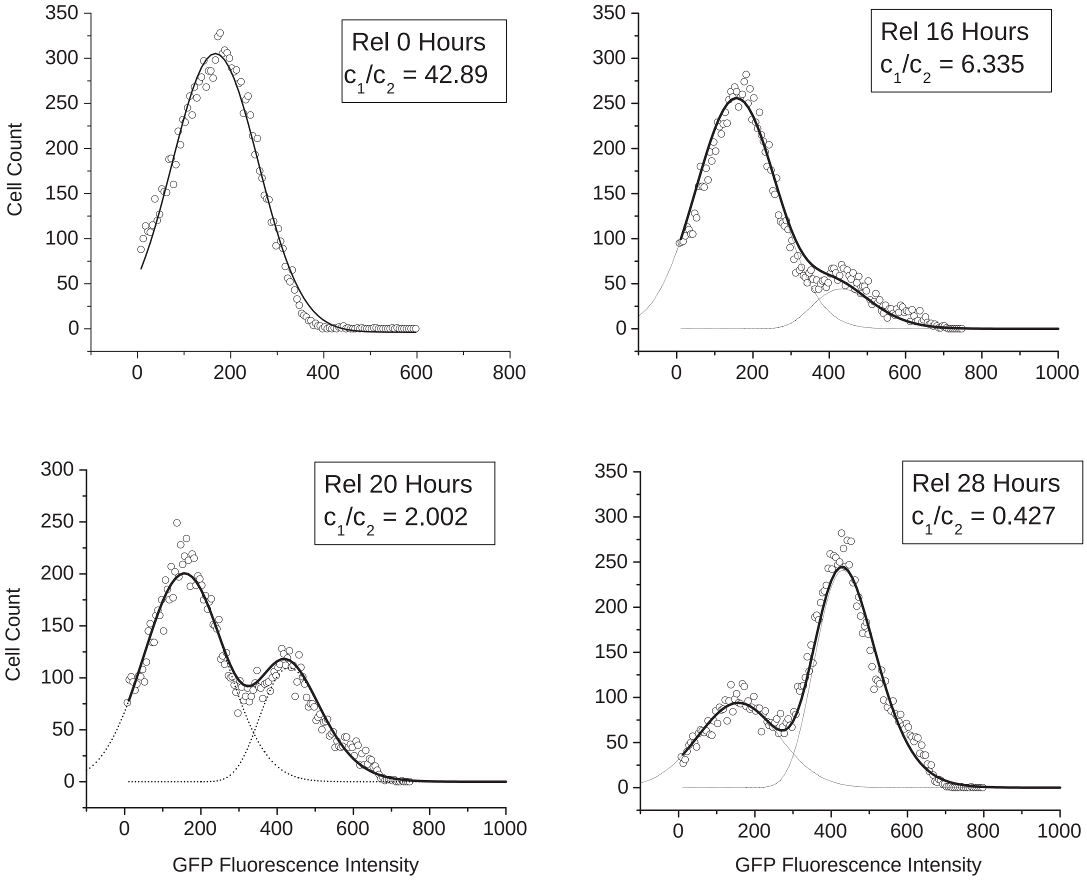

Figure S3: Experimental data for cell count versus GFP fluorescence intensity at selected time points when *gfp* is fused with *rel* promoter. The solid curve represents $P(x,t)$ in Eq. (5) and the dotted curves are the individual terms on the r.h.s. The different parameters of $P_1(x)$ and $P_2(x)$ have the values $x_{01} = 157.14748$, $w_{01} = 150.43575$, $x_{02} = 6.10036$, $w_{02} = 0.1847$ when *gfp* is fused with *rel*.

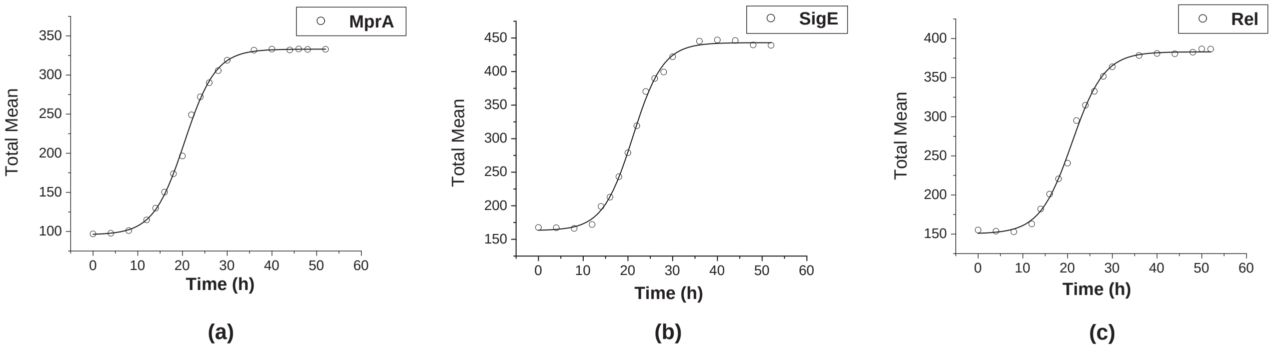

Figure S4: Mean GFP fluorescence level for the total population versus time in the three cases of *gfp* fused with the promoters of (a) *mprA*, (b) *sigE* and (c) *rel*.

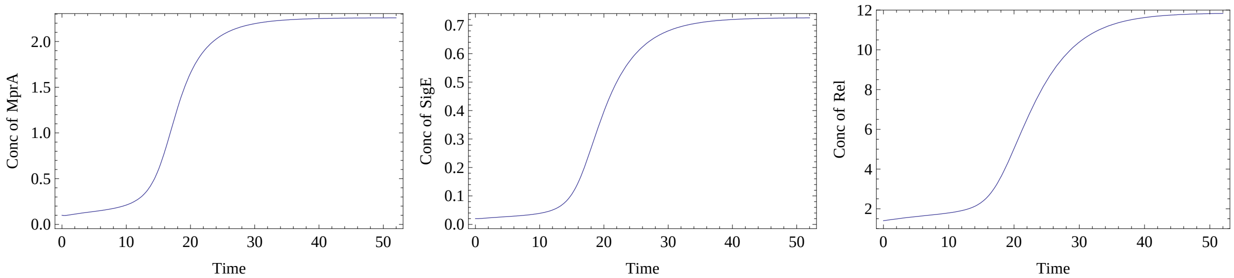

Figure S5: Concentration of MprA, SigE and GFP versus time. The values of the concentrations are obtained by solving Eqs. (6)-(14) in Text S1.

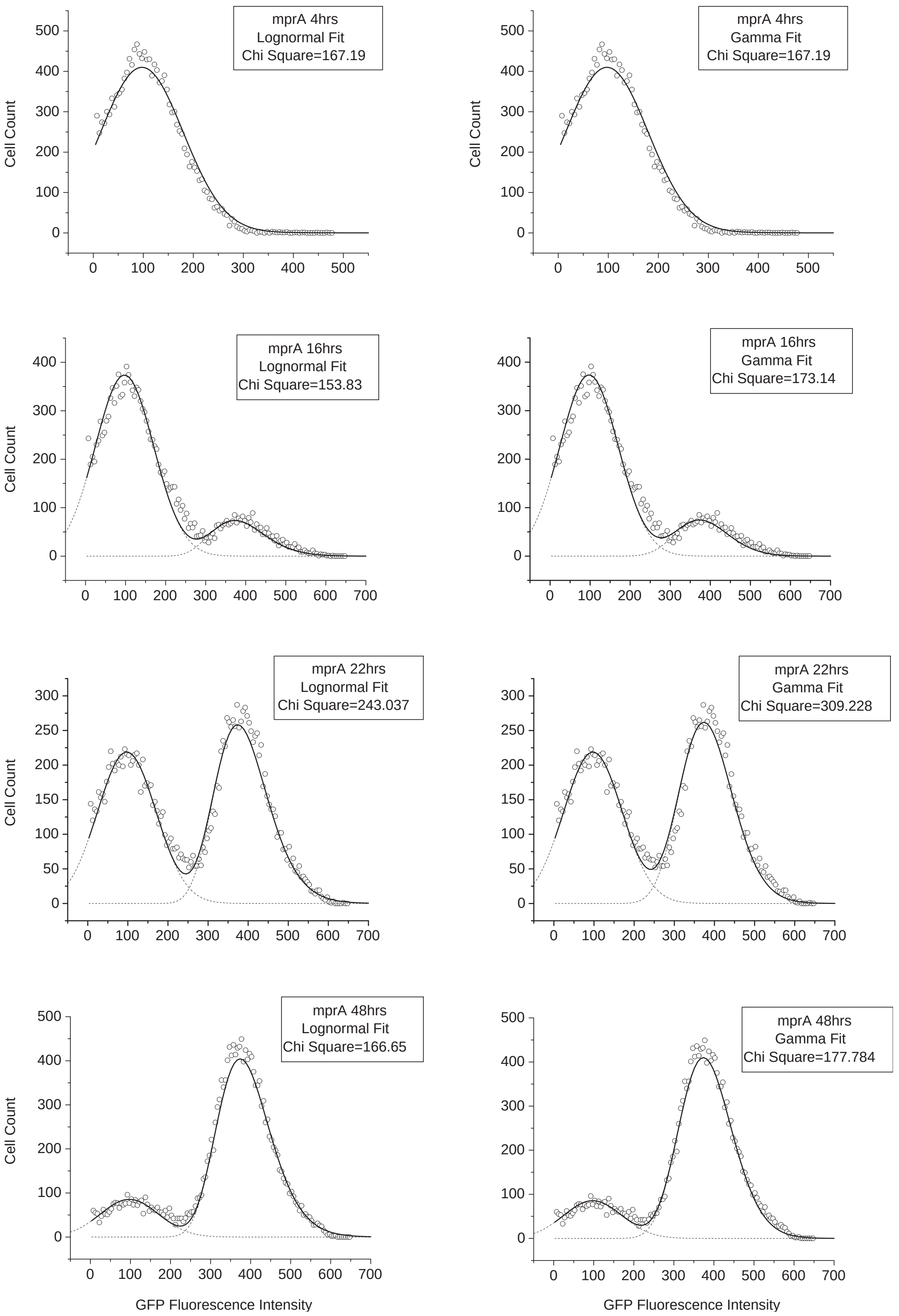

Figure S6: Comparison of fits of experimental data for cell count versus GFP fluorescence intensity at selected time points when *gfp* is fused with *mprA* promoter, with lognormal (Eq. (7)) and gamma distributions. The gamma distribution has the form $P(x) = \frac{x^{a-1}\, exp(-\frac{x}{b})}{b^a\, \Gamma(a)}$, where the parameters a and b have the values $a = 33.246$, $b = 11.59$ and $\Gamma(a)$ is the gamma function.

References

[1] Sureka K, Ghosh B, Dasgupta A, Basu J, Kundu M and Bose I : **Positive feedback and noise activate the stringent response regulator Rel in mycobacteria.** *PLoS ONE* 2008, 3: e 1771

[2] Sureka K, Dey S, Datta P, Singh AK, Dasgupta A, et al. : **Polyphosphate kinase is involved in stress-induced mprAB-sigE-rel signaling in mycobacteria.** *Mol Microbiol* 2007, 65: 261-276.

[3] Zahrt TC, Wozniak C, Jones D, Trevett A : **Functional analysis of the Mycobacterium tuberculosis MprAB two-component signal transduction system.** *Infect Immun* 2003, 71: 6962-6870.

[4] Dahl JL, Kraus CN, Boshoff HIM, Doan B, Foley K, et al. : **The role of RelMtb-mediated adaptation to stationary phase in long-term persistence of Mycobacterium tuberculosis in mice.** *Proc Natl Acad Sci USA* 2003, 100: 10026-10031.

[5] Tan C, Marguet P and You L : **Emergent bistability by a growth-modulating positive feedback circuit.** *Nat. Chem. Biol.* 2009, 5 : 842-848.

[6] Ferrell JE Jr. : **Self-perpetuating states in signal transduction: positive feedback, double-negative feedback and bistability.** *Curr. Opin. Cell Biol.* 2002, 14 : 140-148.

[7] Veening J-W, Smits WK and Kuipers OP : **Bistability, epigenetics and bet-hedging in bacteria.** *Annu. Rev. Microbiol.* 2008, 62 : 193-210.

[8] Pomerening JR : **Uncovering mechanisms of bistability in biological systems.** *Curr. Opin. Biotechnol.* 2008 19 : 381-8.

[9] He H, Zahrt TC : **Identification and characterization of a regulatory sequence recognized by Mycobacterium tuberculosis persistence regulator.** *MprA. J Bacteriol* 2005, 187: 202-212.
